# Near-Infrared Spectroscopy Allows for Monitoring of Bone Fracture Healing via Changes in Oxygenation

**DOI:** 10.3390/jfb15120384

**Published:** 2024-12-19

**Authors:** Cedric Nowicki, Bergita Ganse

**Affiliations:** 1 Innovative Implant Development (Fracture Healing), Departments and Institutes of Surgery, Saarland University, 66421 Homburg, Germany; 2 Department of Trauma, Hand and Reconstructive Surgery, Departments and Institutes of Surgery, Saarland University, 66421 Homburg, Germany

**Keywords:** angiogenesis, microcirculation, perfusion, NIRS, nonunion, bone fracture, vascularization, digital health, intelligent implant, monitoring

## Abstract

Bone fractures are associated with hypoxia, but no longitudinal studies of perfusion measurements in human patients have been reported despite the clinical and research potential. In this longitudinal observational cohort study, the near-infrared spectroscopy (NIRS) device PortaMon was used to assess oxy-(O_2_Hb), deoxy-(HHb) and total (tHb) haemoglobin, as well as the differences between O_2_Hb and HHb (Hb_Diff_) and the tissue saturation index (TSI) at three different depths in the fracture gap. Linear mixed effect models were fitted to analyse time effects. One-way ANOVAs were conducted to compare groups. The time points corresponding to minima were calculated via linear regression. In this study, 11 patients with tibial shaft fractures underwent longitudinal measurements. Additionally, 9 patients with diagnosed tibial shaft nonunion and 23 age-matched controls were measured once. In the longitudinal group, all fractures healed, and decreases in O_2_Hb and Hb_Diff_ (all *p* < 0.05) were observed, with minima occurring 19–21 days after fracture. O_2_Hb values in nonunion patients did not differ from the minima in longitudinally measured union patients, whereas differences in HHb and tHb were significant (all *p* < 0.05). Previously, the onset of hypoxia has been assumed to be much faster. The characteristic trajectories of the NIRS parameters O_2_Hb and Hb_Diff_ can be used to fulfil the need for a non-invasive method to monitor fracture healing. These results suggest that NIRS could supplement radiographs and clinical impressions in daily clinical practice and may enable earlier diagnosis of nonunion.

## 1. Introduction

Bone fractures are associated with hypoxia caused by both an interruption of the bone vascular system and damage to the surrounding soft tissue further inhibiting the oxygen supply [1]. Surgical interventions further reduce the blood flow to the fractured bone [2]. The complex bone vascular system consists of a central artery in the diaphyseal bone marrow that is connected to afferent and efferent blood vessels; a microvascular network with anastomoses, including two capillary networks and the lacunar-canicular system in the compact bone; and sinusoids in the bone marrow [1,3,4]. The onset of hypoxia is thought to occur immediately after fracture or osteotomy upon destruction of the vasculature [5,6,7]. Notably, transverse osteotomies of the femur in rats led to a reduction in total bone blood flow of approximately 50% and a reduction in cortical blood flow in the diaphysis of approximately 40% [8]. However, in the same study, the total bone blood flow doubled four weeks into the healing process. Revascularisation occurs several weeks into healing. Indeed, invasive longitudinal animal studies in rabbits and dogs have shown increases in bone blood flow [9,10,11]. These increases are thought to be mediated by the expression of nitric oxide (NO), hypoxia-inducible factor (HIF) and vascular endothelium-derived growth factor (VEGF), among other factors, which initiate vasodilation and angiogenesis [12]. Blood flow during fracture healing is correlated with the extent of newly formed bone [13]. This connection seems to be determined by metabolic coupling between angiogenesis and osteogenesis [14].

Five to ten percent of bone fractures fail to heal [15], resulting in nonunion, which is associated with high socioeconomic costs [16,17]. Despite the formation of new blood vessels, lower perfusion has been observed in nonunion patients compared to that in union patients; this difference may be associated with the insufficient bone formation found in many nonunion patients [18]. In daily clinical practice, fracture healing is usually monitored based on both clinical appearance and X-ray-based imaging, which has the disadvantages of radiation exposure and providing only a delayed representation of the healing process due to the relatively late onset of calcification [19]. It is often uncertain whether the fracture will heal or not for an extended period; therefore, a common approach is to wait and observe [20]. For clinical practice, it would therefore be advantageous if non-invasive and easy-to-use methods were available to monitor fracture healing and thereby determine patients at risk of nonunion. Currently, no such technology is available for clinical practice. Such a method would also be beneficial for research, i.e., when studying interventions suggested to accelerate fracture healing [21] or the in vivo effects of smart and active implants [22]. Such smart implant systems could allow for continuous monitoring of the progress of fracture healing, and this would allow earlier or even autonomous initiation of interventions as soon as healing delays are identified.

Near-infrared spectroscopy (NIRS) can be used to monitor bone haemodynamics and metabolism in vivo [23], and the NIRS hardware is small enough to potentially be embedded in an implant. It measures three haemoglobin concentrations: oxygenated (O_2_Hb), deoxygenated (HHb) and total (tHb). Haemoglobin is an iron-containing protein that transports gases such as oxygen and carbon dioxide in erythrocytes. Thus, NIRS has been applied to measure decreases in oxygenation in the tibia of healthy human participants with arterial occlusion, as well as to measure increases in oxygenation upon leg elevation [24,25,26]. For example, increases in human tibial tHb were found using NIRS after sublingual application of nitrous oxide (NO) [27]. Additionally, active vasoconstriction in response to sympathoexcitatory stimuli was detected by NIRS in human tibiae [28]. After 14 days of head-down bed rest, Hedge et al. [29] reported slower desaturation kinetics and a lower tissue saturation index (TSI) measured with NIRS in human tibiae. However, no changes in tibial NIRS parameters were observed after exercise compared with rest, and owing to the slow metabolism of bone, high levels of loading and exercise volume seem to be required until metabolic changes can be measured in bone with NIRS [30].

As NIRS can reliably and non-invasively measure several haemodynamic parameters in bone, and, to date, no NIRS data have been published from patients with bone fractures, we tested the following hypotheses regarding measurements in tibial shaft fractures throughout the fracture healing process: (1) low initial values of O_2_Hb will be followed by a linear increase in O_2_Hb with angiogenesis beginning two weeks after fracture. (2) The initial increase in HHb due to hypoxic conditions will decrease with angiogenesis. (3) Decreases in tHb will be observed in the initial healing process when the fracture haematoma is resorbed, followed by an increase with revascularisation. (4) A decrease in the difference between O_2_Hb and HHb (Hb_Diff_) will be observed because of the mechanisms mentioned above. (5) A low TSI will be followed by an increase with revascularisation. (6) No difference will be found between the lowest O_2_Hb values in the longitudinal fracture data and those in the tibial shaft nonunion data, indicating a remaining lack of oxygenation.

## 2. Materials and Methods

Ethical approval for this clinical observational study was obtained from the Saarland Medical Board institutional review board (Ärztekammer des Saarlandes, Germany; application number 127/22). Written informed consent was obtained from participants according to the Declaration of Helsinki prior to the start of the measurements. The study was prospectively registered with the German Clinical Trials Register (DRKS00031942). Participation in this purely observational study did not affect treatment.

### 2.1. Participants and Patients

**Figure 1:**
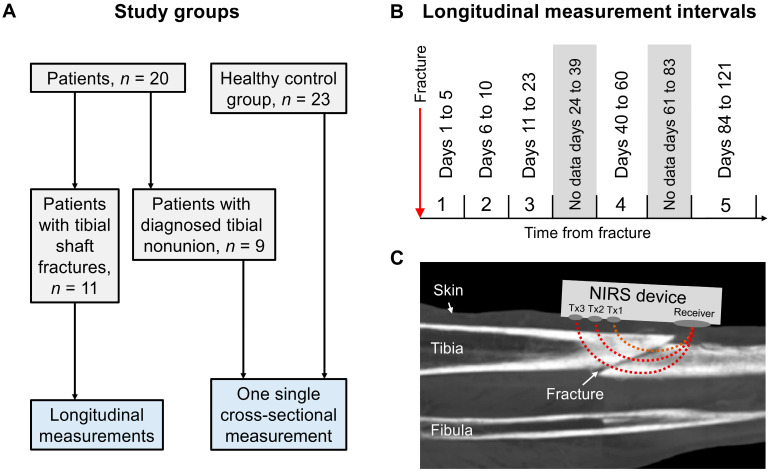
(**A**) Flow chart showing the study groups. (**B**) Time intervals used in the longitudinal analyses. The first measurements were conducted while the patients were still recovering at the hospital. After discharge, patients returned for outpatient visits at approximately two, three and six weeks and three months after the fracture, which resulted in gaps between days 24 and 39 and between days 61 and 83. (**C**) Illustration based on a computed tomography (CT) scan of the lower leg that depicts how the measurements were taken. Tx1, Tx2 and Tx3 are the three individual pairs of LEDs of the PortaMon device. Due to their differing distances from the receiver, they measure at different depths. The exact depths for bone are unknown.

Three study groups were included (Figure 1A): a healthy control group aged 18 years and older whose intact tibial shaft was measured once; a cross-sectional cohort of tibial nonunion patients 18 years and older recruited upon nonunion diagnosis for a single measurement when they presented to the outpatient clinic at Saarland University Hospital; and a longitudinal cohort of patients aged 18 years and older with tibial shaft fractures recruited during the first days of their inpatient stay at Saarland University Hospital between October 2023 and April 2024. The inclusion criteria of the healthy control group were age 18 years and older, no previous fracture or surgery of the legs and no current participation in another study. The inclusion criteria of the cross-sectional tibial nonunion group were age 18 years and older and diagnosed tibial nonunion at least 6 months after the fracture event that has not yet been revised. The inclusion criteria for the longitudinal cohort were 18 years and older, newly obtained fracture of the tibial shaft and no additional more serious fractures or injuries. In addition, all groups had to fulfil the inclusion criterion of the ability for written and verbal informed consent. The exclusion criteria for all three groups were age under 18 years, inability to give written and verbal informed consent and the inability to conduct the measurements at the relevant site.

For the longitudinal cohort, follow-up measurements were scheduled every two to three days during their in-hospital stay and every time they returned to the outpatient clinic. Return visits to the outpatient clinic were generally conducted at approximately two, three and six weeks after surgery; however, the exact timing varied among patients. The authors considered this frequency of measurements sufficient for testing the hypotheses. The frequency of visits to the outpatient clinic determined the time intervals used for data analysis as illustrated in Figure 1B. The implant type used and the time of surgery were not considered in the present analysis, as multiple types of plates and nails were applied, making subgroup analyses impossible.

### 2.2. Measurements

The wearable Bluetooth continuous wave NIRS device PortaMon (Artinis Medical Systems, Elst, The Netherlands) was used to measure O_2_Hb, HHb and tHb in the fracture gap (Figure 1C). Hb_Diff_ was computed as the difference between O_2_Hb and HHb. In addition, the PortaMon device calculates the TSI, a measure of local oxygenation, which is assessed by spatially resolved spectroscopy (SRS). The PortaMon device weighs 75 g, including the battery, and is 9.3 cm by 5.2 cm in size. Data are transmitted by Bluetooth through the PortaSync MKII device (Artinis Medical Systems, Elst, The Netherlands). The PortaMon device records data at a sampling rate of 10 Hz and uses three pairs of LEDs called Tx1, Tx2 and Tx3, with wavelengths of 760 and 850 nm. The detector is a photo diode. The distances between the transmitters and the receiver are 30, 35 and 40 mm. Data were recorded for 10 s and averaged. The total time needed for each patient was around 10 min. Notably, oxygenated and deoxygenated haemoglobin have different absorption spectra for near-infrared light. Therefore, when near-infrared light is transmitted into the tissue and the reflected light intensity is measured, the relative concentration changes of oxygenated and deoxygenated haemoglobin are determined by the Lambert–Beer law. The PortaMon device measures changes in the oxygenation of haemoglobin, primarily in small vessels (<1 mm in diameter), such as capillaries, arterioles and venules, and myoglobin [31]. PortaMon was chosen for this study, as it is among the most studied wearable NIRS devices for measuring skeletal muscle [32], and its small size allows easy use in clinical practice and research alike; moreover, there is currently no device available specifically for measurements in bone. Probes were covered in single-use polyurethane bags and fixed on the skin non-invasively with black kinesiology tape to control for ambient light and to standardize the contact pressure. If needed, the fracture location was confirmed by ultrasound imaging. If a metal plate was implanted, measurements were conducted next to the location to guarantee measurements of the actual fractured bone.

### 2.3. Statistics

Given the lack of comparable human data in the literature, an a priori sample size calculation could not be conducted. The patient sample size was therefore determined by the number of patients available during the 6-month test period. NIRS data were exported via the software Oxysoft version 3.2.72 ×64 (Artinis Medical Systems, Elst, The Netherlands). All the statistical tests were conducted in IBM SPSS Statistics version 30 (IBM SPSS Statistics, Armonk, NY, USA). Significance was defined as *p* < 0.05. The normality of the data was tested with the Kolmogorov-Smirnov and Shapiro-Wilk tests. In the case of nonnormality, the data were transformed using the natural logarithm (ln) to achieve normality. Linear mixed effect models were fitted to analyse time effects with time as a fixed effect and patient as a random effect; a Bonferroni adjustment was made for multiple comparisons. Univariate analyses of variance (ANOVAs) were conducted with post hoc Bonferroni correction to compare groups. The time points at which minima occurred were determined via linear regression using the following equation based on Y = aX + b:

Day of minimum = (b before − b after)/(a after − a before)(1)

## 3. Results

Twenty patients (11 longitudinal tibial fracture patients and 9 patients with tibial nonunion) and 23 healthy control participants were included in this study. The patient and participant characteristics are shown in Table 1. All fractures in the longitudinal group healed. There was no difference in age between the groups (Table 1). The control group data showed no age effects (O_2_Hb: Tx1 *p* = 0.527, Tx2 *p* = 0.684, Tx3 *p* = 0.588; HHb Tx1 *p* = 0.853, Tx2 *p* = 0.672, Tx3 *p* = 0.797; tHb Tx1 *p* = 0.711, Tx2 *p* = 0.767, Tx3 *p* = 0.790; Hb_Diff_ Tx1 *p* = 0.383, Tx2 *p* = 0.400, Tx3 *p* = 0.507; TSI *p* = 0.144). No group differences were found for Hb_Diff_ or TSI. Compared with the fracture and nonunion groups, the control group presented significantly lower O_2_Hb, HHb and tHb values (Table 1).

### 3.1. Longitudinal Data

**Figure 2:**
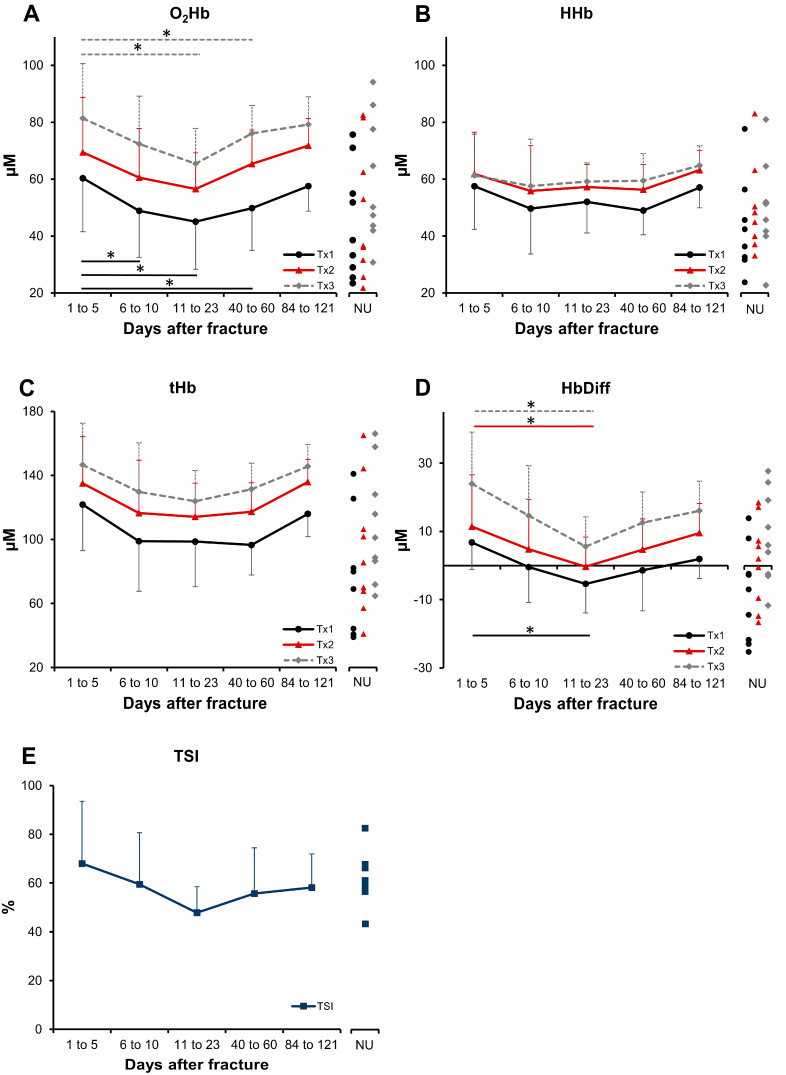
Measurements of (**A**) O_2_Hb, (**B**) HHb, (**C**) tHb, (**D**) Hb_Diff_ and (**E**) TSI. Averages and standard deviations (error bars) are shown. NU indicates nonunion. To the right of each figure, individual data points are displayed for nonunion patients. Differences between time intervals (linear mixed effect model): * *p* < 0.05.

For the longitudinal fracture data, the linear mixed effect model revealed significant decreases in O_2_Hb and Hb_Diff_ over time, but no significant changes were found for the other parameters (Figure 2). O_2_Hb and Hb_Diff_ had their minima between days 19 and 21 after fracture, as indicated in Table 2. The O_2_Hb and Hb_Diff_ values slowly decreased until reaching their minimum values and then increased; notably, this process did not occur abruptly and immediately, as hypothesized. A similar pattern was found for tHb and TSI, but these changes were not significant.

### 3.2. Nonunion

The data from the nonunion patients are shown in Figure 2 to the right of the longitudinal data for each parameter. As shown in Table 1, significant differences between the longitudinal fracture minimum and the nonunion group value were found for HHb and tHb. The difference to the minimum in the longitudinal O_2_Hb data was not significant, neither for Tx1 or Tx2 nor for Tx3 (Table 1).

## 4. Discussion

This is the first study to present longitudinal NIRS perfusion data from human bone fractures. The main findings are as follows: The O_2_Hb values did not immediately reach a minimum value but rather gradually decreased over a period of approximately three weeks. There were no changes in HHb or tHb during healing. A decrease in Hb_Diff_ was observed, followed by an increase after a minimum at approximately three weeks. The TSI did not change. No difference in O_2_Hb values between tibial shaft nonunion compared with the minimum in the longitudinal fracture data was found.

As indicated, the present data revealed a slow decline in fracture gap oxygenation, with the minimum value occurring at approximately three weeks after injury. This finding is surprising, as a much faster onset of hypoxia has previously been assumed and animal studies have reported a drop in oxygen immediately or during the first day [5,6,7]. These studies were, however, conducted in animals and with invasive probes and not with an optical, non-invasive method such as NIRS. The findings of this study seem to indicate that at least some amount of blood supply is provided to the injury site despite vascular damage. This supply could originate from the periphery and periosteum, as well as from blood flowing through the central artery in the bone. These findings are in line with the observation of very slow changes in NIRS parameters when a healthy participant is exposed to high exercise volume and loading [30]. An increase in metabolism along with inflammation and remodelling of the fracture area should increase the oxygen demand. Importantly, the O_2_Hb value determined by NIRS is a measure of oxygen extraction, as gas exchange with the tissue decreases this parameter.

pO_2_, which is 20.9 kPa in the atmosphere, has been determined to be approximately 4.2 kPa in compact bone and between 5.4 and 7.2 kPa in the medullary canal [12,33]. In addition, a steep pO_2_ gradient between the diaphysis and metaphysis was found in rabbit tibial epiphyses from 14.5 to 2.6 kPa, whereas regular arterial pO_2_ is approximately 12–13 kPa [34]. Similarly, the local oxygen concentration in bone determined by two-photon phosphorescence lifetime microscopy is low (<32 mm Hg), despite the high vascular density, with the lowest oxygen concentration of ∼9.9 mm Hg in deeper perisinusoidal regions [33]. Here, the endosteal region was less hypoxic, as it was perfused with additional small arteries. These findings indicate that regular oxygen levels are already low in nonfractured bone. pO_2_ decreases to 1.5 kPa immediately after tibial fractures in mice injury [7]. Bone fracture healing seems to occur independently of oxygen via oxidative phosphorylation and glycolysis, at least during hypoxia and until revascularization occurs [12]. Skeletal stem and progenitor cells (SSPCs) function adequately in hypoxic environments by using glucose and amino acid metabolism in a cell-specific manner [35].

Given this evidence, nonunion may not be caused by a lack of oxygen but rather by insufficient removal of CO_2_, lactate and other waste products and potentially by deficiencies in anaerobic metabolism and the interplay of cellular and growth factors that prevent sufficient angiogenesis and revascularisation. Menger et al. [36], in their review on the vascularization paradox of nonunion formation, noted that there is evidence that vascularization is only one piece of the puzzle in the much more complex process of bone regeneration. This notion is consistent with the findings of the present study, which revealed no difference in O_2_Hb in nonunion patients compared with the minimum longitudinal trajectory in fractures that developed union. This finding indicates that, in nonunion patients, adequate reoxygenation does not occur. In the regular healing process, cytokines and growth fractures induce vascularization of the fracture callus within 2 to 5 weeks [37]. However, the actual oxygenation within the callus tissue of nonunions, as measured in mice by photoacoustic imaging, is impaired despite the presence of blood vessels in nonunion tissue biopsies [37]. This finding supports the assumption that the metabolic coupling between angiogenesis and osteogenesis may depend not only on oxygen availability but also on the removal of waste and the supply of other materials required to rebuild the vasculature and bone, as well as the functions of cellular processes [13,14]. In addition, structural differences were shown in fracture haematomas with delayed healing, where thinner fibres and denser clot structures have been reported [38]. However, it must be acknowledged that different types of nonunion likely have distinct underlying pathological mechanisms. In 34 patients with aseptic nonunion and infected nonunion, the wash-in rate of a contrast agent in contrast-enhanced ultrasound decreased with aseptic nonunion and increased with infected nonunion [18]. The several known types of nonunion have different underlying causes; for example, atrophic and hypertrophic nonunion are caused by either physiological (e.g., lack of blood supply) or biomechanical (e.g., instability and excess forces) factors. The present study did not distinguish between nonunion types, but future longitudinal NIRS studies with a larger cohort of nonunion patients should analyse the different nonunion types separately. In addition, implant types and the time until and from surgery are factors that may affect fracture gap perfusion during the healing process and that should be studied in detail.

The lack of observed changes in HHb, tHb or TSI over time in the longitudinal data of this study, despite a tendency towards decreases in these parameters, may be due to the low number of patients and follow-up measurements. Future studies with a larger patient group may clarify this circumstance. Additionally, the fact that Hb_Diff_ showed the same trajectory as O_2_Hb is certainly because O_2_Hb was used to calculate Hb_Diff_. However, the finding that the control group had significantly lower O_2_Hb, HHb and tHb values than the fracture and nonunion groups was unexpected. This could be explained by the hyperaemia in the surrounding tissues that occurs following injury. As indicated in Figure 1C, NIRS does not measure in the tissue at a specific depth but covers all the tissues intersecting the trajectories of the light coming from the three pairs of LEDs. Another possible cause may be the swelling of the soft tissues in the fracture group. Also, adipose tissue thickness is well known to greatly influence NIRS measurements. As the soft tissues may influence the data, a longitudinal NIRS study should be conducted where the NIRS device is directly attached to the bone, such as in a smart implant. The three separate pairs of LEDs of the PortaMon device recorded data in three different depths, with Tx1 delivering the least deep and Tx3 delivering the deepest readings (Figure 1C). The exact penetration depth of the LED light emitted by the PortaMon device has not been reported for human bone. The most superficial LEDs, Tx1, measured the lowest values in all parameters, while Tx3 showed the highest values. For O_2_Hb, this finding is in line with the fact that pO2, which is 20.9 kPa in the atmosphere, has been determined to be approximately 4.2 kPa in compact bone and between 5.4 and 7.2 kPa in the medullary canal [12,33]. If Tx1 mainly reflects the oxygenation of the cortical bone and the LEDs that measure deeper increasingly assess the oxygenation of the medullary canal, this may be an explanation for the results in this study.

**Figure 3:**
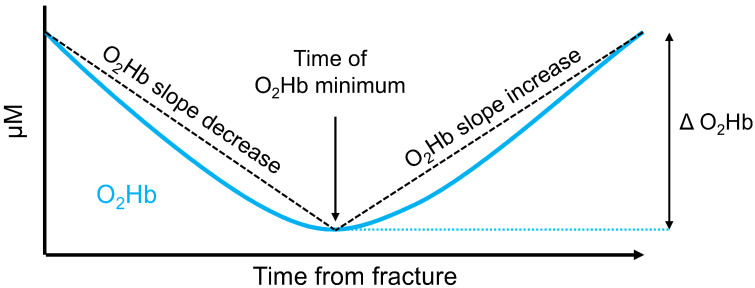
Suggestions of parameters that could be used to monitor fracture healing progress or to assess fracture healing speed in intervention studies. Similar parameters could be computed for Hb_Diff_, but since Hb_Diff_ is calculated on the basis of O_2_Hb, there is no likely benefit of using both parameters at once.

In clinical practice and research, the trajectory of O_2_Hb and consequently Hb_Diff_ shown in this study may be used to monitor progress in fracture healing. The slope of the decrease to the minimum value and the increase after the minimum value, the time to reach the minimum value and possibly also the change in the value of the minimum value compared with the initial O_2_Hb value may be useful parameters (Figure 3). Once O_2_Hb values increase after having previously been lower, union is likely. In cases where O_2_Hb values decrease again, attention must be given to possible complications leading to nonunion. The treatment of malunion and nonunion can be difficult and can take several months to years, with interventions for improving fracture healing being either invasive or non-invasive. The invasive options include fracture revision with a bone graft [39] or the dynamization of an implant, which results in greater forces and movement in the fracture gap [40]. In some cases, the implant can be exchanged or augmented. These invasive modifications that require additional surgery or may be conducted by a smart implant with an actor function can improve the biomechanical conditions for fracture healing and thereby improve the healing process. Non-invasive options with evidence to improve bone healing include pulsed electromagnetic stimulation [41], low-intensity pulsed ultrasound [42] and extracorporeal shockwave therapy [43]. If oxygenation delays are identified earlier, these invasive and non-invasive interventions can be applied sooner, leading to earlier healing success. This is likely to reduce health care costs, decrease immobilization and improve patients’ quality of life [16,44].

Another method that may be used to non-invasively measure changes in fracture microcirculation is laser Doppler combined with white-light spectroscopy [45,46]. This technology measures different parameters than NIRS does, namely, SO_2_, rHb, and blood flow. Thus, combining both technologies to gain more insight into local pathophysiology may be interesting. Apart from perfusion measurement, another non-invasive method to monitor fracture healing that may be suitable for clinical application in an implant or wearable is based on vibration and on changes in signal waveform of the percussion-note with time [47]. Similarly, direct electromagnetic coupling was suggested as a non-invasive monitoring option [48]. However, it required the application of load on the fracture and implant. In addition, among the invasive methods, electrical impedance spectroscopy (EIS) has been used in a smart fracture implant in mice [49]. For clinical use and for use in a smart implant, devices should be ideally small and easy to handle. Results must be displayed immediately after the measurements without requiring further data analyses to ensure the usefulness of such devices in clinical practice. Further studies should ideally provide daily measurements of bone perfusion and oxygenation throughout fracture healing in human patients to determine the precise minima and to learn more about individual variation during healing and circadian rhythms, as well as age effects and effects of smoking and diseases such as diabetes. Further relevant factors that may influence bone oxygenation and that should be studied are sex, medication intake, activity level, fracture type, implant type, the extent of soft tissue injury and the extent of the inflammatory response caused by the injury. Importantly, these studies should be conducted in humans and not in animals, as the findings of this study revealed differences in the trajectory of oxygenation compared with that reported in animal studies [5,7]. In addition, how increases in radiological parameters and fracture stiffness correlate with the NIRS parameters and trajectories throughout healing needs to be studied. However, the available scores based on radiographs, including the RUST-score, are lacking in precision. More quantitative measurements, such as pQCT, would be a possibility, despite radiation exposure of the patients. In addition, the calcification of bone lags behind the clinically relevant increases in fracture stiffness. Unfortunately, stiffness measurements were not possible in the present study.

The main limitations of the present study are its small sample size and infrequent measurements. The measurement times were predetermined by the study design, which provided for a measurement every time a patient returned to the hospital outpatient clinic. The reason for the variation in the exact days of return visits was that the patients were free to arrange the appointment around the intended date. This is common practice in Germany. In addition, the outpatient clinic is only available on weekdays and not on weekends or public holidays. In this study, patients did not receive financial reimbursement for their study participation. In future studies, more frequent measurements could be arranged by reimbursing the patients’ travel costs and maybe by even paying them an additional amount to increase their willingness to participate. In this case, additional insurance cover may be needed. Another weakness of this study is that due to the small sample size, the influence of the implant type on the values and trajectories of the outcome parameters was not analysed. This may be relevant, as intramedullary nailing, other than plating, destroys the central artery in the bone medullary canal [50]. Despite this fact, healing time is shorter in nailing compared with locked plating [51]. The faster healing might be related to biomechanical factors, such as the fact that full weight bearing is possible earlier in nailing than in plating. In addition, factors other than age could have affected the data and should be controlled for in future, larger studies. Factors with a relevant influence of bone oxygenation may be patient sex, medications, pre-existing diseases, activity levels, and others. Such factors should ideally be considered when matching the groups in the future.

Longitudinal perfusion parameter trajectories of patients who develop nonunion could help to determine parameters that can be used to predict nonunion earlier in clinical practice. To determine the viability of such a method, prospective longitudinal studies large enough to include sufficient patients who will develop nonunion are needed urgently.

## 5. Conclusions

This is the first study to present longitudinal NIRS perfusion data from human bone fractures. A slow decline in fracture gap oxygenation was found, with the minimum occurring at approximately three weeks after fracture. This finding is surprising, as a much faster onset of hypoxia has previously been assumed and reported in animal studies. There were no changes in HHb or tHb during healing. A decrease in Hb_Diff_ was observed, followed by an increase after a minimum at approximately three weeks. The TSI did not change. No difference in O_2_Hb values between tibial shaft nonunion compared with the minimum in the longitudinal fracture data was found. The trajectory of O_2_Hb and consequently Hb_Diff_ shown here may be used to monitor fracture healing progress in clinical practice and research. This technology could be integrated in smart implants or wearables to continuously monitor fracture healing. Given the need for an easy-to-use, non-invasive method to monitor fracture healing, the present results suggest that NIRS could be a great complement to radiographs and clinical impressions in daily clinical practice and may even enable early diagnosis of nonunion.

## Figures and Tables

**Table 1:** Patient and participant characteristics and average perfusion parameter values of the fracture, nonunion and healthy control groups. For the fracture group, as the data were longitudinal, the following values were used: O_2_Hb: minimum, HHb: average, tHb: average, Hb_Diff_: minimum, and TSI: average. *p* values are shown in bold when significant. Tx1, Tx2 and Tx3 are the individual pairs of LEDs of the PortaMon device that reflect different depths in the tissue.

Parameter	Fracture Group	Nonunion Group	Control Group	*p* Value Fracture vs. Nonunion	*p* Value Nonunion vs. Control	*p* Value Fracture vs. Control
Total *n* (female, male)	11 (3, 8)	9 (3, 6)	23 (14, 9)			
Age (mean ± SD) in years	60.91 ± 20.84	52.11 ± 19.88	58.74 ± 20.40	0.350	0.412	0.775
O_2_Hb Tx1 (mean ± SD)	45.08 ± 16.77	31.83 ± 24.30	10.51 ± 7.42	0.144	**0.005**	**<0.001**
O_2_Hb Tx2 (mean ± SD)	56.55 ± 12.70	47.70 ± 23.79	27.71 ± 7.63	0.308	**0.002**	**<0.001**
O_2_Hb Tx3 (mean ± SD)	65.45 ± 12.40	59.86 ± 22.19	39.98 ± 8.16	0.566	**0.001**	**<0.001**
HHb Tx1 (mean ± SD)	53.23 ± 13.44	39.45 ± 20.04	20.27 ± 5.22	**0.010**	**0.002**	**<0.001**
HHb Tx2 (mean ± SD)	58.80 ± 12.73	46.67 ± 18.86	28.12 ± 5.40	**0.016**	**<0.001**	**<0.001**
HHb Tx3 (mean ± SD)	60.02 ± 12.60	50.26 ± 16.32	32.96 ± 5.22	**0.042**	**<0.001**	**<0.001**
tHb Tx1 (mean ± SD)	107.62 ± 28.53	71.29 ± 42.48	30.71 ± 11.31	**0.002**	**<0.001**	**<0.001**
tHb Tx2 (mean ± SD)	124.09 ± 27.42	94.56 ± 41.04	55.42 ± 11.80	**0.007**	**<0.001**	**<0.001**
tHb Tx3 (mean ± SD)	135.99 ± 25.16	110.20 ± 36.35	72.41 ± 12.14	**0.009**	**<0.001**	**<0.001**
Hb_Diff_ Tx1 (mean ± SD)	−5.37 ± 8.49	−7.62 ± 13.38	−9.69 ± 5.98	0.664	0.546	0.104
Hb_Diff_ Tx2 (mean ± SD)	−0.34 ± 7.09	1.21 ± 12.80	−0.32 ± 5.72	0.745	0.991	0.641
Hb_Diff_ Tx3 (mean ± SD)	5.56 ± 8.71	9.78 ± 13.90	8.03 ± 8.31	0.433	0.662	0.445
TSI (mean ± SD)	59.56 ± 21.15	62.94 ± 10.65	67.29 ± 6.88	0.642	0.180	0.091

**Table 2:** For those parameters where the difference from the minimum was significant in the linear mixed effect model, the time points at which minima occurred were calculated using linear regression with the following equation: Y = aX + b (linear regression formula); minimum = (b before − b after)/(a after − a before).

Parameter	Regression Before and Including Interval with Expected Minimum	Regression After and Including Interval of Expected Minimum	Calculated Day of Minimum
O_2_Hb Tx1	−0.5008x + 66.286	0.7117x + 40.661	21.13
O_2_Hb Tx3	−0.5851x + 89.096	0.9337x + 59.135	19.73
Hb_Diff_ Tx1	−0.2988x + 8.1601	0.5836x − 9.6608	20.20
Hb_Diff_ Tx2	−0.3285x + 13.741	0.6559x − 4.6142	18.65
Hb_Diff_ Tx3	−0.4924x + 27.466	0.768x + 0.7665	21.18

## Data Availability

The data set analysed during the present study is available from the corresponding author on reasonable request. Access may be granted based on a collaboration agreement. The requesting institution needs to fall within the eligibility criteria of German data protection law.
